# Endogenization of a Prosimian Retrovirus during Lemur Evolution

**DOI:** 10.3390/v13030383

**Published:** 2021-02-27

**Authors:** Kathleen Apakupakul, Sharon L. Deem, Rabia Maqsood, Peeti Sithiyopasakul, David Wang, Efrem S. Lim

**Affiliations:** 1 Institute for Conservation Medicine, Saint Louis Zoo, Saint Louis, MO 63110, USA; apakupakul@stlzoo.org (K.A.); deem@stlzoo.org (S.L.D.); 2 Center for Fundamental and Applied Microbiomes, The Biodesign Institute, Arizona State University, Tempe, AZ 85287, USA; Rabia.Maqsood@asu.edu; 3 Department of Molecular Microbiology, Washington University School of Medicine, Saint Louis, MO 63110, USA; peeti.sithiyopasakul@wustl.edu (P.S.); davewang@wustl.edu (D.W.); 4 School of Life Sciences, Arizona State University, Tempe, AZ 85287, USA

**Keywords:** endogenous retrovirus, gamma-type betaretrovirus, PSRV1, *Propithecus coquereli*

## Abstract

Studies of viruses that coevolved with lemurs provide an opportunity to understand the basal traits of primate viruses and provide an evolutionary context for host-virus interactions. Germline integration of endogenous retroviruses (ERVs) are fossil evidence of past infections. Hence, characterization of novel ERVs provides insight into the ancient precursors of extant viruses and the evolutionary history of their hosts. Here, we report the discovery of a novel endogenous retrovirus present in the genome of a lemur, Coquerel’s sifaka (*Propithecus coquereli*). Using next-generation sequencing, we identified and characterized the complete genome sequence of a retrovirus, named prosimian retrovirus 1 (PSRV1). Phylogenetic analyses indicate that PSRV1 is a gamma-type betaretrovirus basal to the other primate betaretroviruses and most closely related to simian retroviruses. Molecular clock analysis of PSRV1 long terminal repeat (LTR) sequences estimated the time of endogenization within 4.56 MYA (±2.4 MYA), placing it after the divergence of *Propithecus* species. These results indicate that PSRV1 is an important milestone of lemur evolution during the radiation of the *Propithecus* genus. These findings may have implications for both human and animal health in that the acquisition of a gamma-type *env* gene within an endogenized betaretrovirus could facilitate a cross-species jump between vertebrate class hosts.

## 1. Introduction

The study of diseases in nonhuman primates is of acute interest, particularly with recent heightened concern over the emergence of infectious zoonoses due to accelerated anthropogenically driven environmental changes (e.g., [1,2,3]). While many studies have focused on well-documented pathogens affecting both human and nonhuman animals (e.g., SARS-CoV-1, FIV/SIV/HIV, yellow fever, Ebola, SARS-CoV-2) [4,5], there is less known about the origin and evolution of potential pathogens in certain hosts. Diseases of nonhuman primates are of particular concern owing to how human and nonhuman primate hosts are closely related. For this reason, several efforts (e.g., [6,7,8]) are underway to uncover novel pathogens in nonhuman primates that may pose a potential health threat.

Studies of viruses that evolved with lemurs provide an opportunity to understand the basal traits of primate viruses and provide an evolutionary context for host-virus interactions. Lemurs, which are endemic to the island of Madagascar, evolved in geographic isolation from their mainland counterparts due to continental drift [9,10]. Being basal primates, they are an important reference for primate evolution. There are currently 99 extant lemur species that arose 62 million years ago and then diverged into several groups [11,12,13]. Like their hosts, conceivably some of the microbes of lemurs also have been evolving in isolation from mainland Africa, making them unique evolutionary markers. As an example, the discovery of retroviruses of prosimians has had important implications for other extant retroviruses that infect human and nonhuman primates [14,15]. As such, the viruses of lemurs are likely to have a unique evolutionary history in parallel with the lemurs they infect.

In contrast to mainland primates in which many types of viruses have been identified, there is very little known about lemur viruses. To date, Lim et al. [16] described the first extant exogenous viruses of lemurs, and others have discovered endogenized retrovirus and herpesvirus sequences in lemur genomes [14,17,18,19,20,21]. Retroviruses typically infect somatic cells, but on occasion a retrovirus may infect a host germ cell, resulting in an integration of its proviral genome into germline cells. This can lead to the vertical transmission of the virus into host progeny, provided that the infection does not handicap the host and its genome becomes fixed into the host population [22]. These endogenous retroviruses (ERVs) also may have the ability to replicate and generate further germline insertions by retrotransposition or reinfection [23]. ERVs are of particular interest since evidence of endogenized viral sequences in genomes serve as fossil records of viral infections, representing ancestral sequences of modern retroviruses or their extinct relatives [24]. While most ERV lineages do not become fixed into the host population, it has been shown that some ERVs are capable of some level of expression and replication even after tens of millions of years within the host genome [23,25,26]. ERVs have been found in every vertebrate class [21,27,28] and are fairly common; for example, up to 10% of the human genome is made up of ERVs [29,30]. A study screening 65 host genomes across vertebrates uncovered an abundance of diverse ERVs, demonstrating widespread ERV distribution across Vertebrata [21]. Therefore, it is possible that yet-to-be discovered ERVs exist in several taxa, and that there is a possibility for these ERVs to be expressed and replicate postintegration.

Given that the interaction between ERVs and infecting exogenous retroviruses may play a role in pathogenesis, discovering existing ERVs in basal primates such as lemurs has implications for the evolution of host immune responses and for recombination events that may result in cross-species transmissions, particularly between closely related hosts, and even across vertebrate classes [21,23,24]. In addition, lemur viruses evolving in isolation on Madagascar may reveal novel solutions to problems encountered likewise by counterpart viruses evolving in mainland Africa. Here, we describe a novel ERV present in the genome of a lemur, Coquerel’s sifaka (*Propithecus coquereli*). We use molecular analyses to examine the coevolutionary history of this virus with its lemur host and discuss the conservation implications of ERVs in primates.

## 2. Materials and Methods

### 2.1. Sample Collection

Sera were collected from the Saint Louis Zoo (Saint Louis, MO, USA) collection during September–October 2012 from ring-tailed lemurs (*L. catta*), black lemurs (*E. m. macaco*), a blue-eyed black lemur (*E. macaco flavifrons*), mongoose lemurs (*E. mongoz*), black and white ruffed lemurs (*V. variegata*), and Coquerel’s sifakas (*P. coquereli*) (*n* = 32). Lemurs were manually restrained and a blood sample collected from the femoral vein using a 22–24 gauge needle and 1 mL syringe. Sera were collected from free-living lemurs in Madagascar from black and white ruffed lemurs (*V. variegata*), diademed sifakas (*P. diadema*), and Indri (*I. indri*) (*n* = 8). Blood collected from individual lemurs did not exceed 1% of body weight (1 mL/100 g). Thirty-six liver samples from six species of lemurs that had died at the Saint Louis Zoo were pulled from the Saint Louis Zoo biobank for integration site analysis. The animal protocols used in this work were evaluated and approved by the Saint Louis Zoo Institutional Animal Care and Use Committee (Protocol 13-09, 23 August 2013).

### 2.2. Next Generation Sequencing Virus Discovery

Sera specimens were diluted 1:2 in PBS, and total nucleic acid was purified using the COBAS Ampliprep instrument (Roche Diagnostics, Indianapolis, IN, USA) according to the manufacturer’s recommendations. Sequence-independent DNA and cDNA (RNA) amplification was performed as previously described [31]. TruSeq DNA libraries (Illumina, San Diego, CA, USA) were purified and size-selected using Agencourt Ampure XP beads (Beckman–Coulter, Brea, CA, USA). Multiplexed libraries were sequenced on the Illumina MiSeq platform (2 × 250 paired-end reads, MiSeq v2 reagent kit) at the Center for Genome Sciences & Systems Biology at Washington University.

To identify novel virus sequences, sequencing reads were analyzed using previously described computational workflows [31,32]. Briefly, Illumina sequencing reads were trimmed to remove adapter sequences, and overlapping paired-end reads were merged and subsequently quality filtered. Filtered reads were mapped to the *Microcebus murinus* RefSeq genome to remove host sequences. Viral sequences were searched against an NCBI viral database using BLAST (BLASTn and BLASTx sequentially).

### 2.3. Sequence and Phylogenetic Analyses

Nucleotide sequences of the full-length genome of PSRV1 (NCBI GenBank accession number MT787217) were aligned with SRV4 (NC_014474), SRV2 (SIV2DCG), and MPMV (SIVMPCG) using MUSCLE [33]. Gaps in the alignment were removed using the trimAl *gappyout* method [34]. Diversity plots were generated with SimPlot [35], employing Hamming distance sliding windows of 250 nt in 20 nt steps.

Phylogenetic trees were constructed from alignments of the Gag, Pol, and Env protein sequences. The best-fit model of protein evolution was determined by ProtTest v 2.4 [36]. Bayesian Markov chain Monte Carlo (MCMC) inference (JTT + I + G) was performed with BEAST v2.5 [37]. A total of 10,000,000 MCMC states were run with a sample frequency set to 1000 and a 25% burn-in period under a lognormal relaxed clock and Yule prior. Convergence and mixing were assessed with Tracer v1.7 [38].

Phylogenies were also constructed from the nucleotide alignments of the mitochondrion *cytochrome B* gene of *E. macaco* (AF081049.1), *E. flavifrons* (AF081050), *E. mongoz* (AF081051), *L. catta* (LCU53575), *V. variegata* (AB371089.1), *M. murinus* (U53572.1), *P. coquereli* (NC_011053.1), *P. diadema* (NC_026084.1), and *I. indri* (AY441455). Phylogenies were constructed by maximum-likelihood (ML) with the HKY85 substitution model in PhyML v3.0 [39]. A discrete γ distribution of 4 rate categories was used to model heterogeneity among sites. Support for ML trees was assessed by 1000 nonparametric bootstraps. Divergence times estimates were overlaid from Perelman et al. [40]. All ERV sequences identified from the gray mouse lemur (*Microcebus murinus*) genome [21] were aligned to PSRV1. The nucleotide alignment of the *pol* gene sequences was used to construct a maximum likelihood phylogeny (GTR) with 1000 nonparametric bootstraps.

### 2.4. PCR Amplification

Standard precautions to avoid end product contamination were taken for all PCR assays, including the use of PCR hoods and maintaining separate areas for PCR set up and analysis. No-template negative controls were interspersed with actual samples. Accuprime Hot Start Taq DNA polymerase (Invitrogen, Grand Island, NY, USA) was used to amplify 5 μL of extracted samples using *cytB* primers described in Lim et al. [16] with the following PCR program: 95 °C for 5 min, 40 cycles of 95 °C for 30 s, 55 °C for 30 s, 72 °C for 29 s, followed by 72 °C for 10 min. The following primer set was used for PSRV1 screening: forward primer 5′-CATCTTAATGCCCAGTCCTTGCGAC-3′ and reverse primer 5′-GATAGACAGGTGGGCTCGTTCCAC-3′, with an expected product size of 457 bp. Products were visualized following electrophoresis on 1.25% agarose gels. Amplicons were purified by QIAquick gel extraction kit (Qiagen, Germantown, MD, USA), cloned into pCR4 using TOPO TA cloning kit (Invitrogen, Grand Island, NY, USA), and clones from multiple transformed bacterial colonies were sequenced to verify their identity.

### 2.5. Integration Analyses

A preliminary genome assembly of Coquereli’s sifaka (Pcoq_1.0) was obtained from Ensembl (Accession GCA_000956105.1). PSRV1 coding sequences were queried against the assembly using megaBlast (1 × 10^−3^), minimum length of 150 nt. For each hit, the nearest gene was determined as annotated by the Ensembl browser.

### 2.6. LTR Analyses

Genomic DNA was extracted from liver tissue using the DNeasy Blood & Tissue kit (Qiagen). Primers were designed to the 5′ long terminal repeat (LTR) using a forward primer annealing to the U3 region (5′-CTGCGGAAGAGCTTGTAAGTTTC-3′), and the reverse primer to Gag (5′-CCAGTACGCAAAGGCAGTCACTGGAAC-3′), and to the 3′ LTR sequences using forward primer annealing to Env (5′-CTCATCAATTAATCTCTGATGTCCAAGC-3′), and the reverse primer to the U5 region (5′-CAAGAAATGGAGACAAGACAGGTCTC-3′). Sequences were amplified using high fidelity Accuprime *Pfx* DNA polymerase (Invitrogen), gel purified and cloned with a TOPO TA cloning kit (Life Technologies). Sequences from at least three bacterial clones per tissue specimen were confirmed by Sanger sequencing. The resulting LTR sequences were aligned using MUSCLE [33] and estimated using a simple rate of evolution 2.8 × 10^−9^ and 4.5 × 10^−9^ substitutions per site per year [14,17,41].

## 3. Results

**Figure 1:**
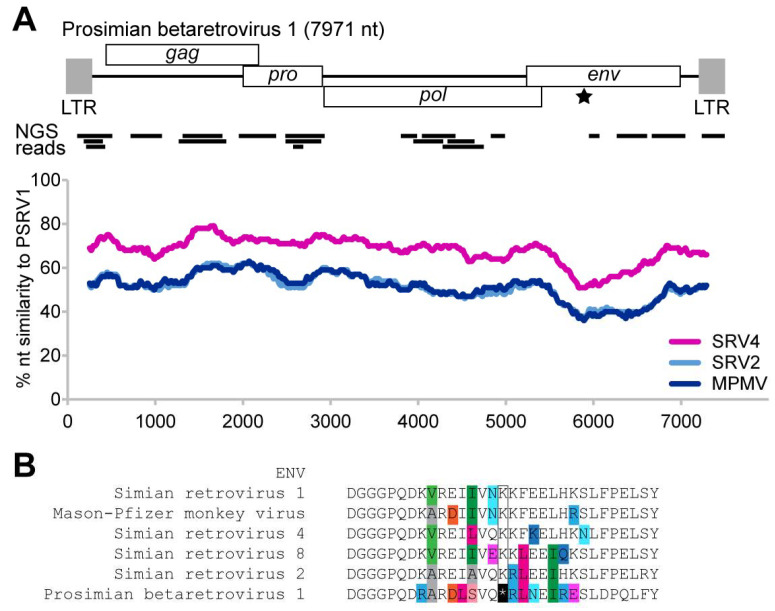
The prosimian retrovirus 1 (PSRV1) genome and its sequence similarity to closely related retroviruses simian retrovirus (SRV) 2, SRV4, and Mason–Pfizer monkey virus (MPMV). (**A**) PSRV1 encodes four open reading frames: *gag*, *pol, pro,* and *env*. A single premature stop codon was found in the *env* gene (marked by a star). Initial Illumina next-generation sequencing (NGS) reads mapping to PSRV genome are indicated in black bars below. (**B**) Sequence alignment of the Env protein shows that the premature stop codon position is otherwise a highly conserved lysine amino acid in other betaretroviruses.

As part of a broad search for viral biodiversity, total nucleic acid extracted from sera specimens of captive lemurs in the Saint Louis Zoo was subjected to next-generation sequencing. Bioinformatic analyses identified 20 sequencing reads with limited identity to primate betaretroviruses (Figure 1A). These sequencing reads were used to design primers to amplify the genome, resulting in a 7900 nt complete genome sequence. The resulting genome sequence was 40–60% similar to simian retrovirus (SRV) 2, SRV 4, and Mason–Pfizer monkey virus (MPMV). Thus, in line with the nomenclature, the lemur retrovirus genome sequence was provisionally named prosimian retrovirus 1 (PSRV1).

The PSRV1 genome is predicted to encode four opening reading frames—Gag, Pol, Pro, and Env. A single premature stop codon was found in the Env protein. Sequence alignment of Env shows that the premature stop codon position is otherwise a highly conserved lysine amino acid in other betaretroviruses (Figure 1B). The 5′ LTR has a Lys1,2 primer binding site.

**Figure 2:**
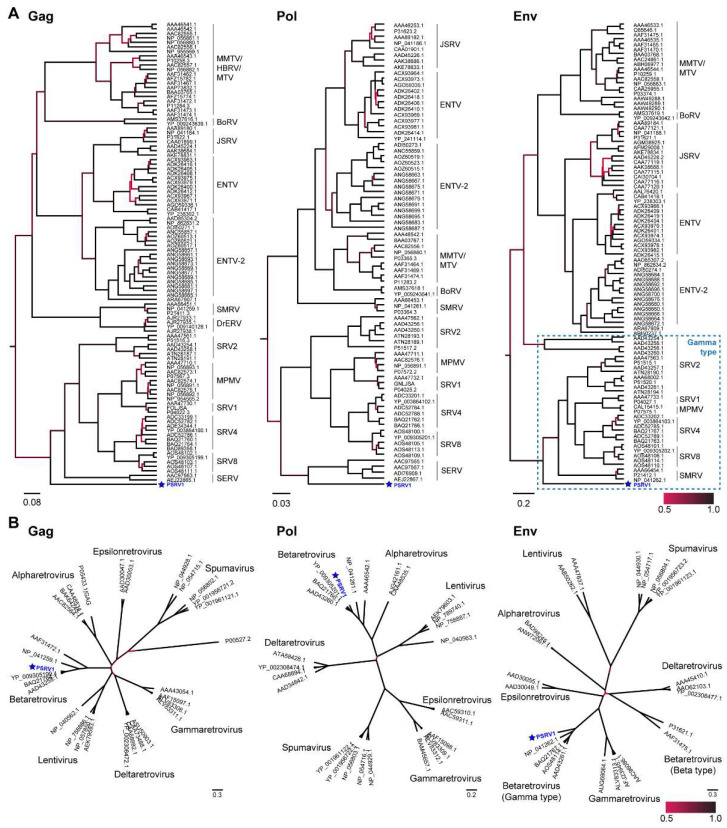
Phylogeny of PSRV1 and closely related retroviruses. (**A**) Phylogenies generated by Bayesian inference of Gag, Pol, and Env sequence alignments. (**B**) Phylogenies of Gag, Pol, and Env protein sequence alignments from PSRV1 and representative genome sequences of genera within the *Retroviridae* family are shown. Scale indicates posterior probability values.

Phylogenetic analyses of the Gag, Pol, and Env proteins indicate that PSRV1 is most closely related to other betaretroviruses (Figure 2A,B). PSRV1 forms a well-supported monophyletic clade with simian betaretrovirus (SRV1, SRV2, MPMV, and others). Due to recombination, there are two classes of *env* gene of betaretroviruses. The gamma-type sequences have a highly conserved CX6C motif, while beta-type sequences have a highly conserved CX4CL motif. The PSRV1 Env has the CX6C motif and falls within the diversity of other gamma-types, indicating that the PSRV1 is most similar to the gamma-type betaretroviruses.

**Figure 3:**
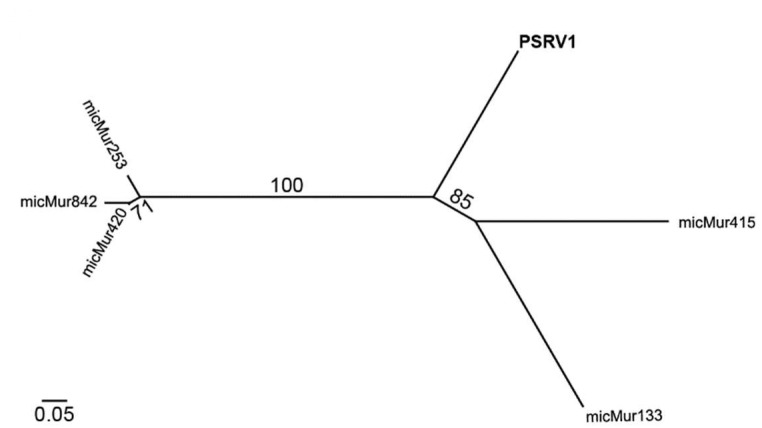
Phylogeny of PSRV1 and partial retrovirus sequences identified in gray mouse lemur genome. Phylogenies generated by maximum likelihood of *pol* gene nucleotide sequence alignment of PSRV1 and alignable retrovirus sequences identified in Hayward et al. [21]. The endogenous retrovirus (ERV) with the highest nucleotide identity to PSRV1 was a region in the *pol* gene from micMur415 sharing 57.78% identity. Scale indicates bootstrap values.

We compared the PSRV1 genome to retroviral sequences identified in the *Microcebus murinus* (gray mouse lemur) genome by Hayward et al. [21]. In contrast to the complete genome sequence of PSRV1, most of the ERV sequences from the *Microcebus murinus* genome were partial fragments or highly mutated resulting in inframe premature stop codons. Only five partial genome sequences were alignable to PSRV1, all of which were identified as putative beta-like ERVs consistent with our findings (Figure 3).

In order to determine the prevalence of the PSRV1 in lemurs, we designed primers in the *pol* region for PCR screening. In addition to the index Coquerel’s sifaka, we screened total nucleic acid extracted from the serum of another two Coquerel’s sifaka. Both sifakas tested positive by PCR, demonstrating that PSRV1 was present in DNA. Additionally, we screened sera from a diverse panel of lemur species from the Saint Louis Zoo collection—2 black lemurs (*Eulemur macaco macaco*), 3 mongoose lemurs (*Eulemur mongoz*), 2 black-and-white ruffed lemurs (*Varecia variegata*), and ringtailed lemurs (*Lemur catta*). No individuals of the other lemur species tested positive for PSRV1.

**Figure 4:**
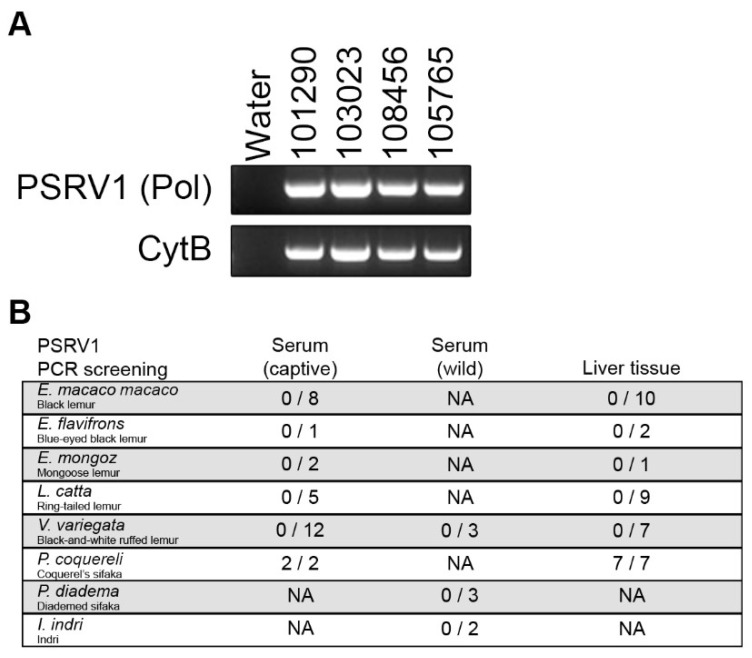
Molecular validation and screening for PSRV1. (**A**) Gel electrophoresis shows PCR amplification of PSRV1 (*pol* gene) and host cytochrome B gene (*cytB*) of nucleic acid extracted from 4 Coquerel’s sifaka liver tissues (IDs: 101290, 103023, 108456 and 105765) and a water negative control. (**B**) Screening results of a diverse panel of lemur species. Only *P. coquereli* tested positive for PSRV1 in both sera and liver tissue.

We next sought to determine the prevalence of PSRV1 in free-living lemurs. Sera collected from lemurs in Madagascar, including the closely related diademed sifaka (*P. diadema*), black-and-white ruffed lemurs (*V. variegata*), and Indri lemurs (*Indri indri*), were screened. Although the *cytB* gene was readily amplified indicating that DNA was present, none of the specimens were positive for PSRV1 (Figure 4).

We hypothesized that PSRV1 could be endogenized in the genome of Coquerel’s sifaka. If this was the case, proviral DNA from germline integration would be detected. Therefore, we isolated total nucleic acid from liver tissue specimens from a panel of lemur species. All Coquerel’s sifaka livers tested, but none from the other species, were PCR-positive for PSRV1. It is possible, although unlikely, that all Coquerel’s sifakas tested, either captive or free-living, would have been infected by the same exogenous virus. However, the more parsimonious explanation is that PSRV1 is endogenized in all Coquerel’s sifakas. Taken together, this suggests that PSRV1 is endogenized in Coquerel’s sifaka but not in the related diademed sifaka.

Using a Coquerel’s sifaka preliminary genome assembly that had been released, we sought to investigate the integration sites of PSRV1 in silico. We identified PSRV1 in 3 scaffolds (KQ021870, KQ024747 and KQ025014) suggesting that there are at least 3 integrated copies of PSRV1 (Table 1).

**Figure 5:**
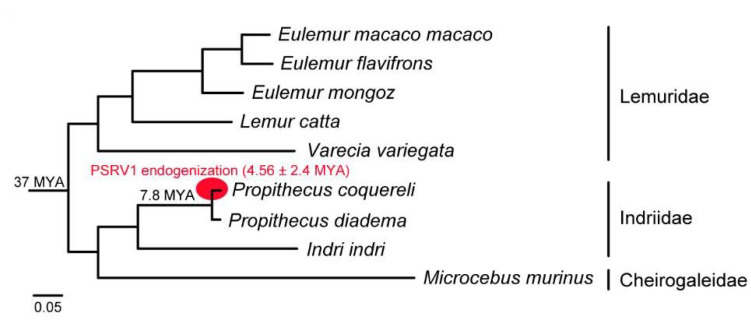
Cladogram of lemur species divergence and PSRV1 endogenization overlay. Estimated time of integration of PSRV1 into *P. coquereli* genome occurred within 4.56 MYA (±2.4 MYA) is indicated in red.

In order to estimate the time of integration, we sought to compare sequence divergence between the 5′ and 3′ LTR as the LTR sequences are identical at the time of integration. We PCR-amplified, cloned, and sequenced the LTRs from two Coquerel’s sifaka. The LTR sequence divergence was 0.0228 substitutions per site. Using a molecular evolutionary rate of 2.5 × 10^6^ sites/year, we estimate that PSRV1 integration occurred within 4.56 MYA (±2.4 MYA). By overlaying the integration time estimate on a phylogeny of lemur speciation, this was consistent with published divergence times (Figure 5). Thus, this indicates that PSRV1 endogenization occurred at least after the split of *Propithecus* spp. 5.1 MYA.

## 4. Discussion

In contrast to mainland primates in which many types of viruses have been identified, there is very little known about viruses that infect lemurs. However, there is an urgent need to characterize their unsampled viral biodiversity as: (1) lemurs are one of the most endangered taxa; therefore, a better understanding of potential health threats is critical to sustaining long-term conservation efforts, and (2) as humans encroach upon wilderness areas, the likelihood for pathogenic viruses to evolve the ability to infect humans increases [42,43,44]. In this study, we tested a phylogenetically diverse array of lemur species from the Saint Louis Zoo as part of a broad search for viral biodiversity. We have characterized a novel lemur retrovirus that has been endogenized in sifaka genomes, which we have provisionally named prosimian retrovirus 1 (PSRV1). PSRV1 DNA was detected in Coquerel’s sifaka liver tissue but not in the other lemur species sampled, suggesting that PSRV1 is specific to *P. coquereli*. Because identical long terminal repeat (LTR) sequences flanking the proviral genome are generated at the time of integration, the time of endogenization can be estimated by comparing the sequence divergence between the 5′ and 3′ LTRs. Therefore, these genomes carry ERV sequences that are believed to represent past infection of germ cells [24]. Sequence divergence estimates date PSRV1 integration into the sifaka genomes to approximately 4.56 million years ago (±2.4 MYA). Our results also suggest that PSRV1 endogenized during the radiation of the *Propithecus* genus (approximately 7.8 MYA) and is consistent with its absence in black, ring-tailed, and black-and-white ruffed lemur genomes (Figure 4B) and with the Lemuridae/Indriidae lineage split 37 MYA (Figure 5). This adds to the growing evidence [21] that lemurs have been exposed to retroviruses for at least 4 million years within Madagascar, and unknown exogenous strains still might be circulating among species in the wild.

We identified PSRV1 as a novel endogenous betaretrovirus basal to other primate betaretroviruses that is most closely related to simian retroviruses, with a genome sequence that is 40–60% similar to Mason–Pfizer monkey virus (MPMV) and simian retroviruses (SRV) 2 and 4 (Figure 1). Other known primate betaretroviruses that cause significant disease include Langur virus and Squirrel monkey retrovirus [45]. Historically, simian betaretrovirus infection can result in high morbidity and mortality, particularly in captive primates [45]. Recent attention has been directed to endogenous lentiviruses due to the implications for lentivirus evolution and for uncovering the origins of HIV [14,17]. Gifford et al. [14] discovered an endogenous lentivirus in the gray mouse lemur, prosimian immunodeficiency virus (pSIVgml), which gave insight into the origins of HIV/SIV as an example of the introduction of a lentivirus into a primate host and their subsequent interaction and evolution. By comparison, despite the detection of an abundance of beta-like ERVs across vertebrate groups [21], primate betaretroviruses remain understudied as, for example, the risk of zoonotic transmission of SRV has yet to be determined [45,46]. In much the same way that interrogating the functions of viral genes from basal primate lentiviruses was critical to identifying human-specific adaptations that human immunodeficiency virus (HIV) acquired after the initial cross-species transmission events [47,48], examining functional genes in other primate viruses also should be prioritized to identify zoonotic risk. Examining the endogenous lentivirus pSIV also provided the opportunity to study the activity of host defense factors in the setting of germ line invasion [49]. Given that betaretroviruses also can induce serious disease in nonhuman primates, determining their zoonotic risk to humans is paramount. This risk is exacerbated by the presence of ecotourist resorts in Madagascar that harbor fully habituated lemurs and allow close human-lemur contact [12].

Other endogenous betaretroviruses have been detected in at least one other species of lemur. Hayward et al. [21] quantified ERV abundance across vertebrates, analyzing conserved regions (Gag, Pro, Pol) across a range of host genomes. They identified multiple ERVs within the genome of gray mouse lemurs. Notably, gray mouse lemurs (Cheirogaleidae) and sifakas (Indriidae) are phylogenetically distinct lemur species with disparate life histories, belonging to family groups that split around 34 MYA at the end of the Eocene [40]. Secondly, based on sequence alignments, none of the ERVs in the gray mouse lemur genome matched PSRV1 (Figure 3) consistent with our results that PSRV1 was not detected in other sister species in the Indriidae subfamily. Third, most of the ERVs identified in the gray mouse lemur genome are were partial fragments or highly mutated resulting in inframe premature stop codons. The open reading frames of PSRV1 are largely intact except for a single premature stop codon in the env gene (Figure 1B). Although we predict that this would lead to a nonfunctional Env protein if expressed, functional studies are needed to demonstrate it. We did not find evidence of PSRV1 expression and cannot exclude the possibility that PSRV1 env may be transcomplemented by other retroviruses. Attempts at identifying the integration sites from sequencing reads were unsuccessful. Understanding the integration site and characterization of copies of PSRV1 genomes will need to be addressed in future studies.

Retroviruses have been found to be extreme host generalists with widespread host-switching among distantly related vertebrates [21]. We identified PSRV1 as a gamma-type betaretrovirus based on Gag, Pol, and Env phylogenies. These types of betaretroviruses are distinct in that the topology of the transmembrane (TM) subunit of the envelope glycoprotein (*env*) gene differs from the respective topology of the highly conserved reverse transcriptase (RT) region of the *pol* gene, on which retrovirus phylogeny is commonly based. This points to a past recombination event in which heterologous *env* sequences were acquired [21,24,50]. Recombination of the *env* gene can effectively alter retrovirus host range of a virus including rescuing the virus [51,52]. The potential host range of PSRV1 is not known. With the current global health threat from infectious diseases, the zoonotic potential of lemur viruses should be a critical concern, particularly in light of how closely related lemurs are to humans and other primates [43,53,54].

In addition, infectious diseases threaten at risk species with extinction [55]. While there have been advances in environmental and habitat conservation efforts of endangered lemurs, there is a strikingly disproportionate gap in our understanding of infectious agents of lemurs. Many lemur species are threatened with extinction due to habitat loss and fragmentation [56], and the burden of infectious agents on lemurs is poorly understood, underscoring the need to characterize unsampled viral biodiversity. The host of PSRV1, *P. coquereli*, is listed as endangered [57] and is particularly susceptible to habitat loss and hunting [12]. The discovery of PSRV1 adds to our catalog of what little is currently known about viruses that infect lemurs, and as more lemur viruses are discovered may provide more insight to the infectious agents currently circulating in their ranges.

The discovery of viruses embedded in host genomes, while not currently known to be infectious, provides a “snapshot” in time of what viruses might have been circulating in the environment at the time of endogenization, and how host-virus interactions may have affected coevolution. Examining endogenous viruses, many of which are the ancient relatives of extant viruses, fills in the gaps between ancient and recent viral evolution [58]. Our study uncovers a unique host-virus interaction in lemur evolutionary history and demonstrates that striking biodiversity can even be found hidden within the genomes of endangered wildlife.

## Figures and Tables

**Table 1:** PSRV1 integration sites in *P. coquereli* preliminary genome sequence assembly.

Scaffold	Genomic Location	Nearest Gene	Distance to Gene	Orientation
KQ021870	Scaffold KQ021870: 2937467-2938067	PLPP2	21.5 kb	Forward
KQ024747	Scaffold KQ024747: 2937467-2938067	NAV1	57.8 kb	Reverse
KQ025014	Scaffold KQ025014: 2937467-2938067	EZH1-201	6.0 kb	Reverse

## Data Availability

The complete genome sequence of PSRV1 has been deposited to the NCBI GenBank database under accession number MT787217.
